# Photodynamic immunotherapy of cancers based on nanotechnology: recent advances and future challenges

**DOI:** 10.1186/s12951-021-00903-7

**Published:** 2021-05-29

**Authors:** Zhaoyuan Liu, Zhongjian Xie, Wenting Li, Xinqiang Wu, Xiaofeng Jiang, Guanhua Li, Liangqi Cao, Dawei Zhang, Qiwen Wang, Ping Xue, Han Zhang

**Affiliations:** 1 Department of Hepatobiliary Surgery, The Second Affiliated Hospital of Guangzhou Medical University, Guangzhou, 510260 China; 2 Department of Traditional Chinese Medicine, The Second Affiliated Hospital of Guangzhou Medical University, Guangzhou, 510260 China; 3 Key Laboratory of Optoelectronic Devices and Systems of Ministry of Education and Guangdong Province, Collaborative Innovation Centre for Optoelectronic Science & Technology, Institute of Microscale Optoelectronics, Shenzhen University, Shenzhen, 518060 China; 4 College of Physics and Optoelectronic Engineering, Shenzhen University, Shenzhen, 518060 China; 5 Shenzhen Key Laboratory of Micro-Nano Photonic Information Technology, Guangdong Laboratory of Artificial Intelligence and Digital Economy (SZ), Shenzhen University, Shenzhen, 518060 China; 6 Department of Cardiovascular Surgery, Sun Yat-Sen Memorial Hospital, Sun Yat-Sen University, Guangzhou, 510120 Guangdong China

**Keywords:** Photodynamic therapy, Nanotechnology, Immunotherapy, Cancer treatment

## Abstract

Photodynamic therapy (PDT) is a non-invasive or minimally-invasive treatment which applies photosensitizers (PSs) to create reactive oxygen species (ROS) exposed to light trigger to destroy cancer cells. PDT can activate host anti-tumor immune responses but not powerful enough to kill metastatic tumors. Because of its carrier advantage, imaging, and therapeutic function together with enhanced permeability and retention (EPR) effect, nano-materials have already been used in photo-immunotherapy. Herein, photodynamic immunotherapy (PDIT) based on nanotechnology seems to be a hopeful new form of cancer therapy. In this article, we firstly summarize the recent development in photodynamic immunotherapy based on nanotechnology.

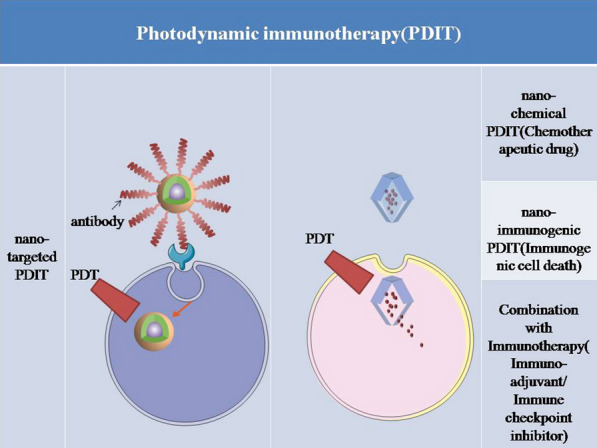

## Background

Cancer still accounts for a primary cause of death across the world. Cancer is characteristic of poor prognosis and high mortality despite systemic therapy such as chemotherapy and radiation. Metastasis and recurrence are two thorny issues in treating cancer. Primary tumors can be treated with surgery, chemotherapy, radiotherapy, and so forth. Unfortunately, metastasis and recurrence will eventually lead to the death of cancer patients [1].

Photodynamic therapy (PDT) uses photosensitizers (PSs), oxygen, and light to destroy tumors through direct cell kill, microvascular disruption, and inflammation [2]. PDT has lower side effects and systemic toxicity than chemotherapy and radiation therapy. PDT has been sanctioned by the Food and Drug Administration of America for clinical use in treating a variety of solid tumors [3].

PDT uses reactive oxygen species (ROS) created by PS under the light trigger to kill tumors. ROS has a very short life span and narrow radius, especially for singlet oxygen [4]. Precise targeting of PDT is critical for delivering photosensitizers to wanted areas to produce ROS. PDT is a very effective treatment for skin lesions and esophageal cancer but can not destroy deep tumors due to the shallow light penetration through tissues [5]. Most photosensitizers for PDT are hydrophobic and suboptimally selective in vivo [6]. To solve these problems, nanomaterials that are hydrophilic and can be used as drug delivery have been conjugated with conventional photosensitizers. Nanomaterials can passively accumulate in tumors via enhanced permeability and retention (EPR) effect [7–10], which is crucial for an effective PDT that minimizes collateral damage to surrounding healthy tissues. Therefore, a large amount of nano-delivery systems have been designed to meet this requirement.

PDT has shown the ability to induce anti-tumor immunity. However, the anti-tumor immune response caused by PDT is usually mild. Tumor hypoxia can inhibit T cells from entering tumor areas to cause immune suppression [11]. PDT has been shown to induce immunogenic cell death (ICD) and release tumor-associated antigens during the destruction of cancer cells, leading to activation and proliferation of CD8^+^ T lymphocytes [12]. However, the therapeutic effect of PDT on metastasis and recurrence is still weak.

Nanomaterials are defined as particles between 1 and 100 nm in size. Traditional organic PSs are hydrophobic, unstable, and unspecific in vivo when applied in PDT. So, nanomaterials with excellent physical and chemical properties have been constructed to solve these problems [13]. Nanomaterials are general terms of 0D, 1D, 2D, and 3D materials with small size effects, which are composed of ultrafine particles with sizes less than 100 nm (0.1–100 nm). According to the geometric structure, nanomaterials include 0D materials (quantum dots), 1D nanomaterials (nanotubes or nanowires), 2D nanomaterials (thin films), and three-dimensional (3D) nanomaterials. The extraordinary interaction of light and matter gives low-dimensional materials the potential for photodynamic therapy. 0D materials are promising nanocarriers for their high emission quantum yield, tunable emission, and facile surface modification. The versatile surface modification of 1D nanomaterials can make them excellent PDT agents. 2D nanomaterials have gained extensive attention for their unprecedented technological advances [14–17]. Many 2D nanomaterials have been constructed and applied in photo-detection [18–20], optical application [21–25], fiber laser [26, 27], environmental science [28, 29] and energy [30]. 2D materials have low toxicity, high stability, good biocompatibility, tunable composition, facile surface modifications, and strong interaction with light. These features are ideal for a variety of PDT applications. 2D black phosphorus has been used in photodynamic therapy and drug delivery [31]. 3D nanomaterials are hydrophobic and exhibit intrinsic toxicity, many modifications need to make to overcome these issues (Table 1) [32–45].

**Table 1:** Nanomaterials applying in PDT

Nanomaterials	Promising properties	Improvements	Toxicity
0D			
Quantum dots(QDs) [32–35]	High emission quantum yield, tunable emission, promising carrier, good biocompatibility, deep PDT	Release of heavy metals from the QD cores	Potential toxic effects
1D			
Carbon nanotubes [36]	Versatile surface modifications, drug delivery	Lack of standardized synthetic protocols	Low toxicity
2D		Get appropriate lateral size with large-scale production, tumor-targeting efficiency, biocompatibility	Low toxicity
Manganese dioxide (MnO_2_) nanosheets [37–39]	Regenerate oxygen, carrier, good biocompatibility		
2D transition metal dichalcogenide (TMD) nanosheets [40]	High specific surface area, good biocompatibility, facile modification, and ultrahigh light and heat conversion efficiencies		
Graphitic-phase carbon nitride (g-C_3_N_4_) nanosheets [41]	High stability, good biocompatibility, and high photoluminescence quantum yields		
Black phosphorus (BP) [42–44]	Exceptional structural, optical and chemical properties, impressive carrier mobility, good biocompatibility and biodegradability inside the human body		
3D			
Fullerenes [45]	Drug delivery	Poorly soluble in water	Intrinsic toxicity

In recent years, cancer immunotherapy has become an effective method to treat various malignant and metastatic tumors. Cancer immunotherapy has shown the ability to destroy residual tumors but often fails to kill primary tumors [46]. Cancer immunotherapy remains restricted to patient benefits and circumscribed anti-tumor efficacy despite significant advances [47]. Nanomaterials can carry immune adjuvants or immunosuppressants to specific sites in the tumor. Therefore, the combination of PDT and immunotherapy based on nanotechnology might be the solution.

## Nano-targeted photodynamic immunotherapy

Nano-targeted Photodynamic immunotherapy is a combination of PDT and molecular-targeted therapy based on nanotechnology. Nano-targeted Photodynamic immunotherapy exhibit better tumor targeting and killing but less toxicity as compared to conventional PDT.

### Tumor-targeted nano-photodynamic immunotherapy

Hepatocellular carcinoma (HCC) is one of the most deadly diseases in the world, but only a little part of patients with HCC are eligible for curative surgery. Therefore, new types of therapy are imperative for the treatment of HCC. The biomarkers of HCC cell surface such as epithelial cell adhesion molecule (EpCAM) [48] and Glypican-3 (GPC3) [49] are amenable for the development of photodynamic immunotherapy based on antibody-photosensitizer conjugate (APC) strategy.

**Fig. 1:**
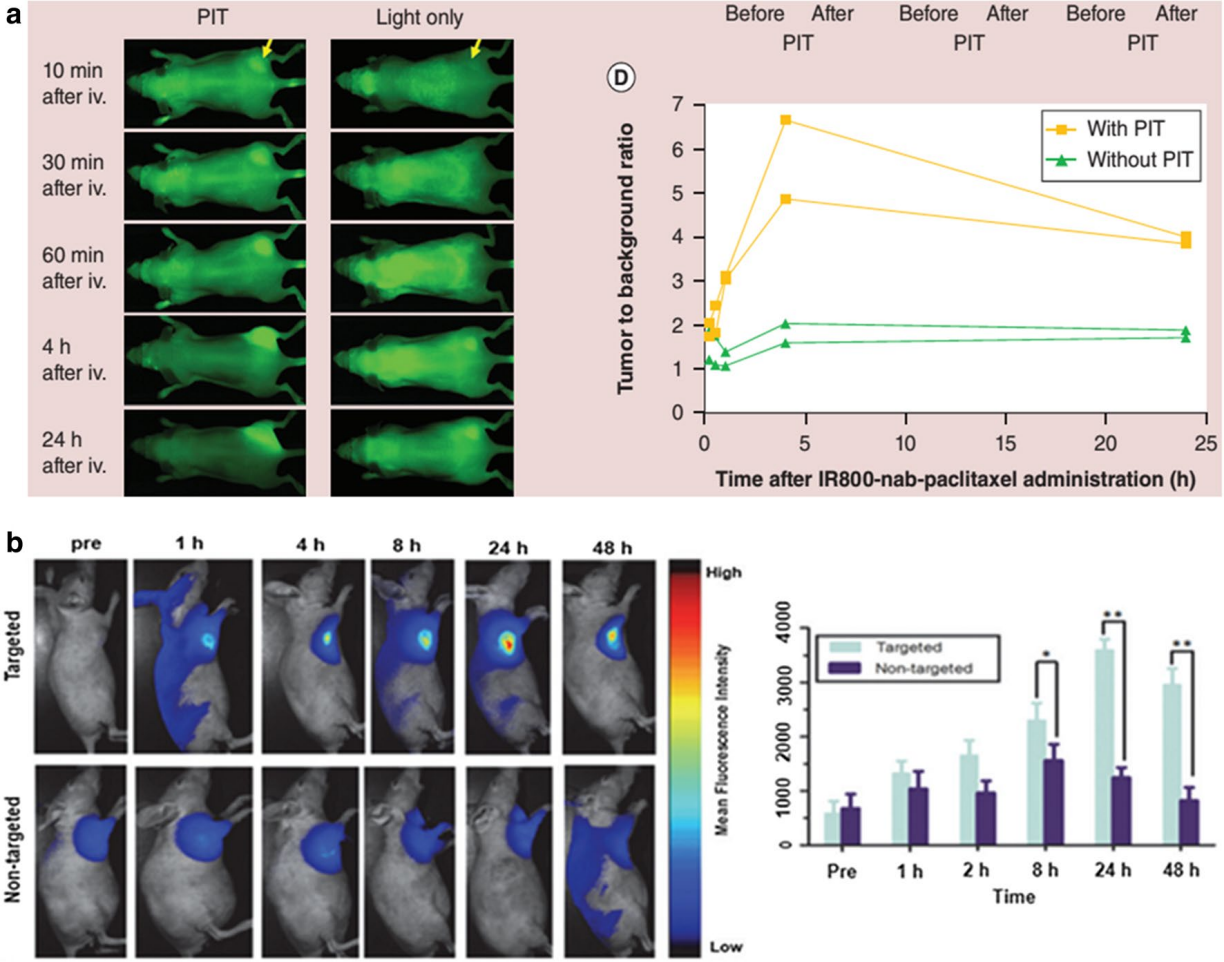
**a** Dynamic images and continuous TBR of IR800-nab-paclitaxel over time for A431/G1 tumors only after IR700-YP7-mediated nuclear or near-infrared irradiation. IR800-nab-paclitaxel had rapid, high accumulations, and high TBR in acute PIT treated tumors compared with untreated tumors [49] Copyright ©2015 Future Science Group. **b** Comparison of mean fluorescence intensity between the anti-EPCam-UPG (targeted) and the UPG micellar (nontargeted) at different time points before and after injection of targeted and nontargeted groups in tumor-bearing mice [48] (Copyright ©2018 Royal Society of Chemistry)

Hanaoka et al. reported that in vitro animal experiments have shown that antibody-targeted photodynamic immune nanomaterials rapidly aggregate in tumor sites. Within 60 min after the injection in animals treated with photodynamic immunotherapy (PIT), the tumor became visible and the target background ratio (TBR) was greater. This showed that IR700-YP7 PIT could significantly increase the accumulation of nano-drugs to the tumor area, thus improving drug efficacy (Fig. 1a) [49]. As shown in Fig. 1b [48], Significant green luminescence was observed in both groups. Besides, anti-EpCAM-UPG micelle treated mice expressed stronger signals, showing excellent in vivo activity targeting ability. Compared with untargeted micelles that showed passive tumor targeting capability only, anti-EpCAM-conjugated micelles had active and passive targeting capabilities, resulting in a higher tumor aggregation rate of micelles in tumors even after 48 h. Compared with the non-targeted group, the mean fluorescence intensity of the targeted group at 8 h, 24 h, and 48 h showed a significant difference. By EPR effect, the fluorescence signal gradually decreased 24 h after injection.

**Fig. 2:**
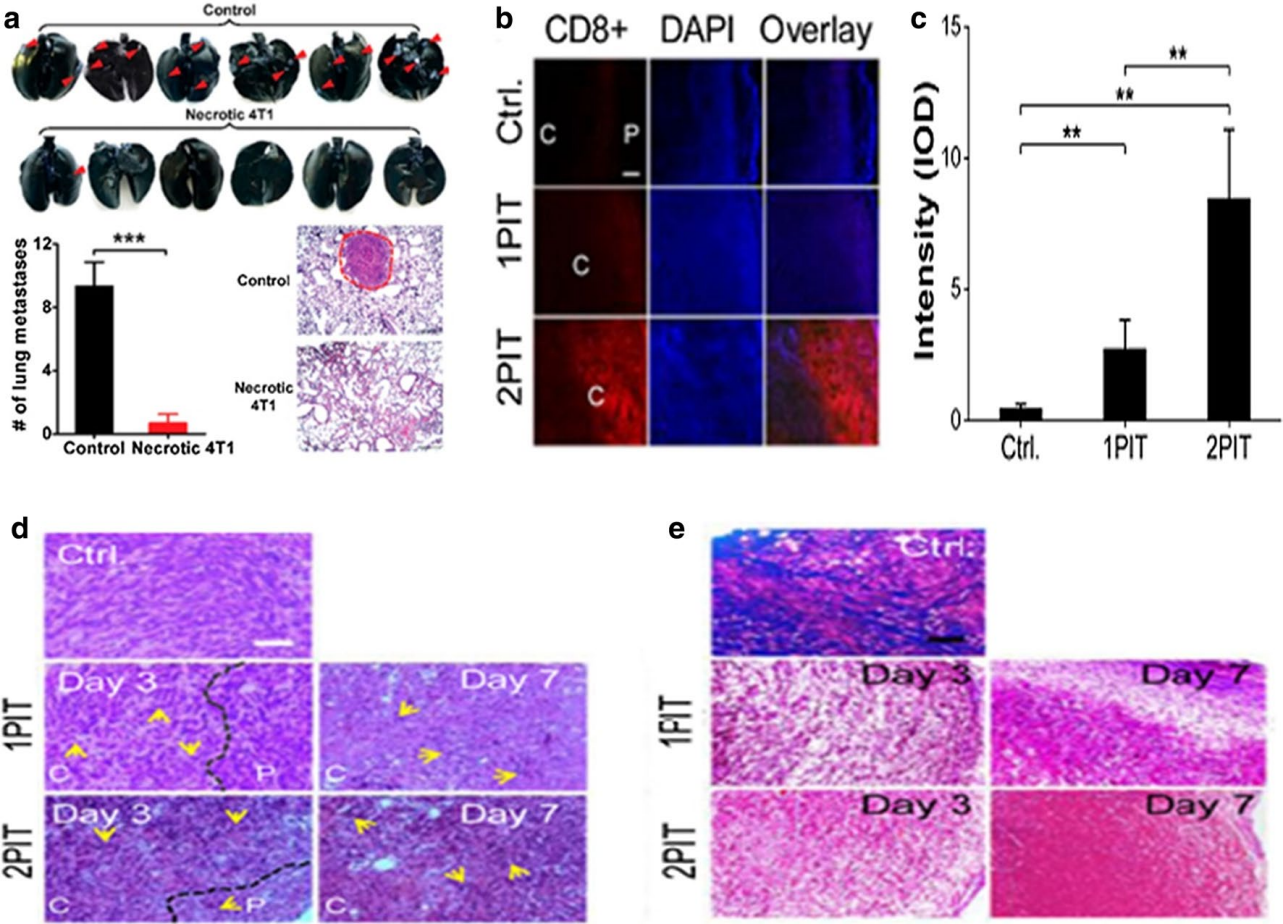
**a** Quantitative analysis of lung metastases and H&E staining of lung tissue. intrapulmonary metastasis showed by arrows[50] Copyright © 2017 American Chemical Society. **b** Anti-CD8a dyeing. "C" & "P" represent central and peripheral areas of the tumor, respectively. **c** Fluorescence intensity-based tumor CD8+ T cell frequency **, P < 0.01. **d** H&E staining of tumor tissue. Yellow arrows, neutrophils. **e** Trichrome staining results. black/blue, nuclei; red, muscle fibers; Blue, collagen [51] (Copyright © 2016 American Chemical Society)

Yu et al. reported that PDT-stimulated anti-tumor immunity is related to the damage of tumor cells. Necrotic tumor cells caused by tumor integrin αvβ6-targeting PDT activated dendritic cells to increase CD8^+^T cell infiltration in the tumor [50]. There were more lung metastases in the control group (Fig. 2a). By comparison, lung metastatic growth was significantly lower in the inoculation group than in the control group (P < 0.001). The tumor microenvironment (TME) could be a new target for enhanced photoimmunotherapy against cancer. Fibroblast-activation protein (FAP) is expressed on carcinoma-associated fibroblast (CAF), which is an important part of tumor extracellular matrix (ECM). Zhen et al. reported that FAP-targeted PDT led to ECM deposition and enhanced T cell infiltration significantly, and then effectively suppress tumor [51]. IF microscopy showed a significant increase in the number of CD8^+^T cells in tumors after one or two doses of PIT (Fig. 2b). Positive staining in tumors was increased by 6.13 times in the 1 dose PIT group and 19.0 times in the 2 dose PIT group compared with the control group (Fig. 2c). At the same time, increased neutrophil infiltration was observed on the H&E staining too (Fig. 2d). In the control group, a lot of cellular islets separated by a thick layer of collagen were observed on tumor slices. After PIT, significant ECM destruction and fewer cell colonies were observed (Fig. 2e).

For large solid tumors such as HCC, tumor-targeted PDT can indeed enhance efficacy, but it is less likely that it will also be effective in the center of the tumor due to the interstitial obstruction of the tumor. Tumor stromal targeted PDT seems to solve this problem.

### Vascular-targeted nano-photodynamic immunotherapy

**Fig. 3:**
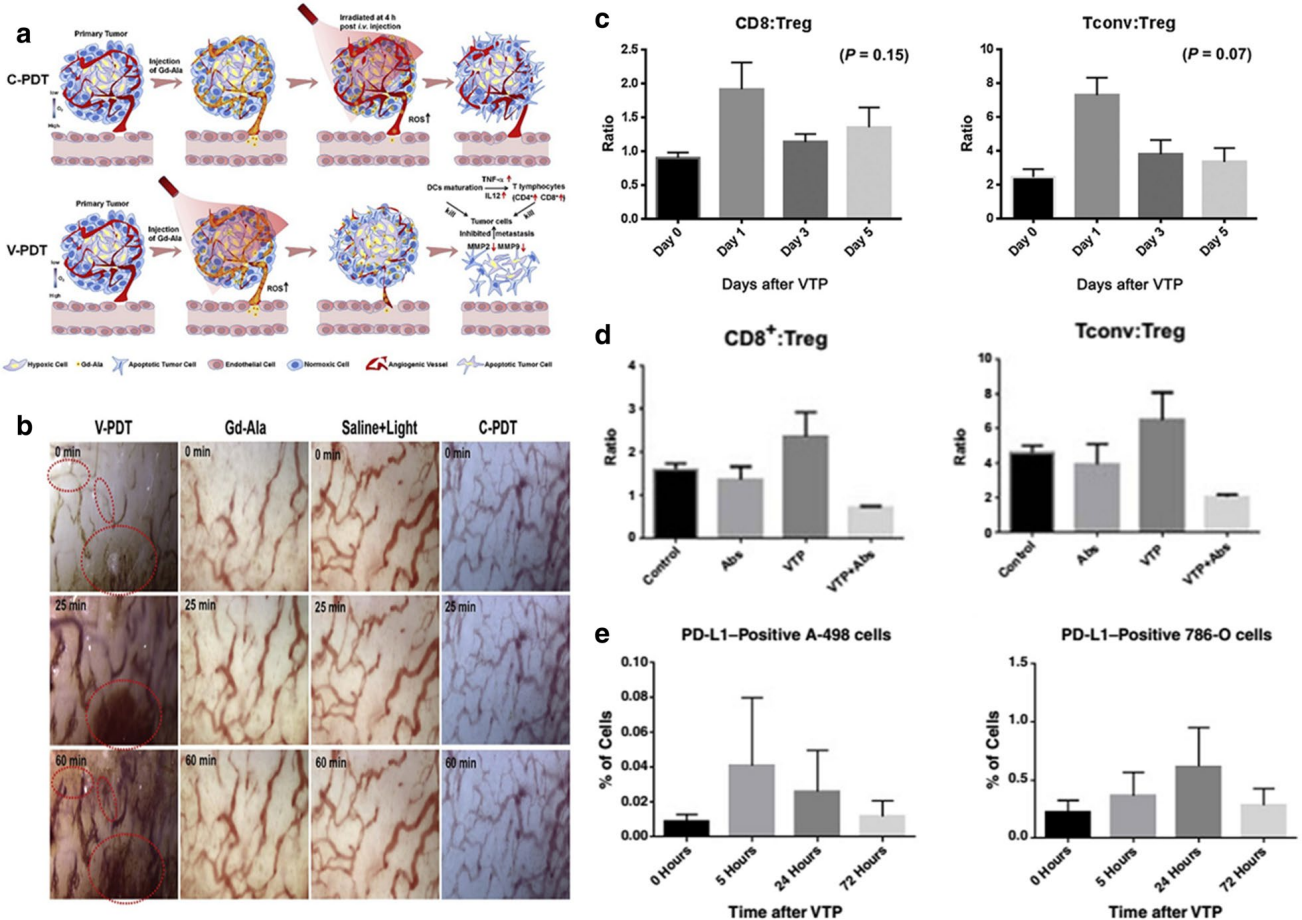
**a** Schematic diagram of cell-oriented PDT(C-PDT) and vascular-targeted PDT (V-PDT). **b** The real-time changes of tumor vasculature undergo different treatment methods[52] Copyright © 2019 Elsevier, Inc. **c** Ratios of CD8+:Treg (P = 0.15) and Tconv:Treg cells (P = 0.07) were calculated. **d** The lungs were taken on the 21st day and the CD8+/Treg and Tconv/Treg cell ratios were calculated. **e** VTP induced expression of PD-L1 in human RCC xenograft tumor [53]. (Copyright © 2017 American Association for Cancer Research)

Oxygen is sufficient in blood vessels, making it a perfect target for PDT by producing ROS, which also destroys vascular endothelial cells causing vessel disruption. Guan et al. reported that tumor vascular destruction PDT promoted dendritic cells (DCs) to mature, and then secreted interleukin-12 (IL-12) and tumor necrosis factor-α (TNF-α), which activate T lymphocytes by upregulating the differentiated clusters of CD4^+^ and CD8^+^ T lymphocytes. Besides, the down-regulation of matrix metalloprotein 2 (MMP2) and MMP9 also reduced the rate of tumor metastasis [52]. First, the nano-gadofullerene (Gd@C82-Ala, abbreviated Gd-Ala) was injected and simultaneously exposed to light. Activated Gd-Ala can utilize the oxygen of blood vessels to create ROS, to block blood vessels via effectively destroying endothelial cells. Then, the activated GD-Ala promoted endothelial cells to maturation, which secreted TNF-α and IL-12, thereby activating T lymphocytes by upregulating CD4^+^ and CD8^+^ T lymphocytes differentiation clusters. Besides, down-regulation of MMP2 and MMP9 reduced tumor metastasis too. Photo-triggered Gd-Ala can destroy tumor blood vessels and inhibit metastatic tumors (Fig. 3a). To understand real-time changes of vessels in the GD-Ala-based V-PDT process, a model of 4T1-Luc tumor cells implanted in the dorsal skinfold chamber (DSFC) was built. The large-scale skin of the mouse is used for angiography, which has high optical clarity. The changes of blood vessels are shown in Fig. 3b.

O'Shaughnessy et al. reported that vascular-targeted photodynamic (VTP) therapy-induced local immune response can enhance an immune response to PD-1 pathway inhibition. The PD-L1 expression can be induced by VTP in renal-cell carcinoma (RCC) xenograft [53]. In the VTP+ antagonistic antibody (Abs) group, CD8^+^ and Tconv cells played a role in combined treatment response. The ratio of CD8:Tregs to Tconv: Tregs returned to baseline 3 days after treatment (Fig. 3c). The ratio of CD8^+^:Treg and Tconv: Treg cells was also dropped in the VTP+ Abs group compared to the control group or monotherapy treated animals (Fig. 3d). The tumor was treated with VTP, then the expression of PD-L1 was quantitatively evaluated. The proportion of cells expressing PD-L1 increased after VTP treatment compared with untreated tumors in both xenograft models. In Both A-498 and 786-O tumors, the expression of PD-L1 peaked 5 h and 24 h after VTP treatment (Fig. 3e).

The VTP can destroy vascular endothelium and cause vascular obstruction. For larger tumors, the tumor blood vessels are larger. Even if the VTP can cause damage to tumor blood vessels, it is relatively mild and cannot block blood vessels. So the treatment of the primary tumor is not as effective as the treatment of the smaller tumor. This is the key problem to be solved in vascular-targeted photodynamic immunotherapy.

## Nano-chemical photodynamic immunotherapy

Administered chemotherapeutic drugs may cause chemosensitivity reduction, drug resistance, and organ toxicities. Nanomaterials can enhance drug stability and increase drug-associated cytotoxicities, while reducing systemic toxicity via releasing encapsulated drugs by triggered of light. Some chemotherapeutic drug like mitoxantrone (MX) is also photosensitizer. Therefore, MX can act as a photosensitizer to generate ^1^O_2_ for PDT and an anti-tumor drug at the same time [48]. Nanosized albumin-bound paclitaxel under targeted PDT also increases in the concentration of paclitaxel within the tumor [49].

**Fig. 4:**
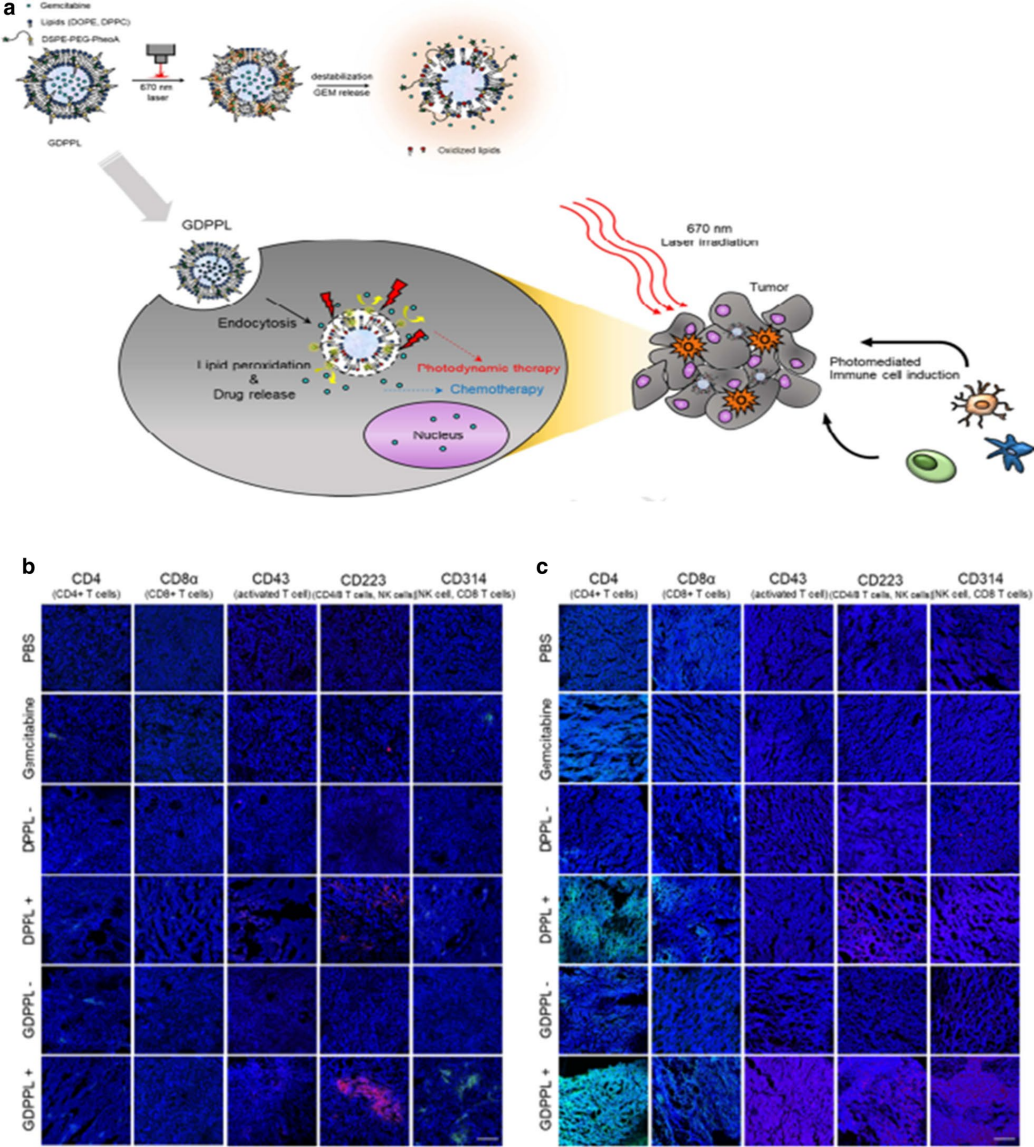
**a** Graphical abstract of Gemcitabine-loaded DSPE-PEG-PheoA liposome-mediated photoimmunotherapy. Immune cells were recruited in vivo to detect tumor sections by PBS-, free GEM-(40 mg/kg), DPPL- or GDPPL (GEM dose; 3 mg/kg and/or PHEOA; **b** BALB/c nude mice and **c** BALB/c mice were irradiated with or without a 670 nm laser (n = 4)(Scale bar = 100 μm)[54] (Copyright © 2018 Elsevier Ltd)

Nano-chemical PDT can enhance immune effects against tumors. Specifically, Kim e al. reported that the release rate of gemcitabine (GEM) due to disruption by ROS produced from PDT was increased. The drug delivery nanoplatform under PDT enhances the number of various immune cells, which are effective anti-tumor immunotherapy for biliary carcinoma (Fig. 4a) [54]. Due to the lack of T lymphocyte production, there was no significant T cell fluorescence observed in BALB/c nude mouse tumors (Fig. 4b). In BALB/c mice, DSPE-PEG-pheophorbide A liposome (DPPL) combined with laser irradiation of GEM-loaded DSPE-PEG-pheophorbide A liposome (GDPPL) stimulated a large number of CD4^+^ and CD8^+^ T cells to infiltrate HUCCT-1 tumor tissue due to exogenous stimulation of photomediated affiliates, while the PBS, DPPL and GDPPL treatment groups did not receive laser irradiation without any immune cells (Fig. 4c).

Chemotherapy at this stage is still effective in many tumors. But like a double-edged sword, its inevitable side effects seriously affect the quality of patients' life. The main reason is that its systemic application may bring inevitable damage to normal tissues and cells. Nanomaterials can accurately deliver chemotherapeutic drugs to the tumor to avoid systemic toxicity and increase the concentration of chemotherapeutic drugs in the tumor. By further use of immune response of chemotherapeutic drugs of nano-PDT, nano-chemical Photodynamic Immunotherapy will give chemotherapy a new life.

## Nano-immunogenic photodynamic immunotherapy

Immunogenic cell death is followed by damage-associated molecular patterns (DAMPs), which serve as immunogenic signals to induce anti-tumor immunity. The immunogenic effects of PDT can be enhanced via nanomaterials to achieve better antitumor immune responses against metastasis of tumors.

**Fig. 5:**
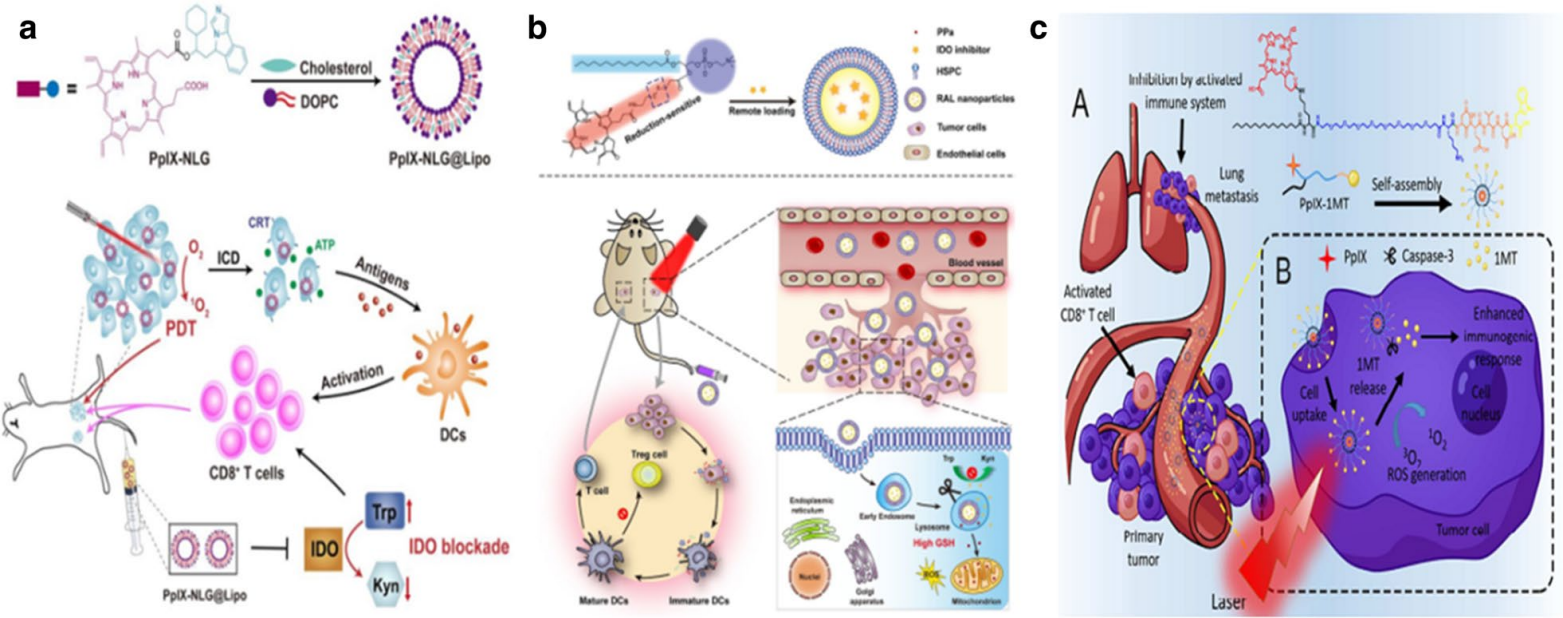
**a** Schematic diagram of PM-PDT in combination with aPD-1 therapy. **b** FACS assay of DC maturation (CD80+CD86+) in different treatments; Matured DCs with different treatments detected by Quantitative analysis(n = 3). **c** Detection of CD8^+^ and CD4^+^ cells by FACS assay[57] (Copyright © 2019 American Chemical Society)

Oxygen-boosted PDT, by applying Fento-like-Haber–Weiss catalyst and MnO_2_ to nanoplatform, can elicit ICD to release DAMPs, which induces immune and effector cell activation. Thus systematic anti-tumor immune response against metastasis was strongly induced [55, 56]. Plasma membrane (PM)-targeted PDT can produce ROS to rupture PM followed by induction of ICD and fast release of DAMPs. Then, DAMPs would enhance anti-tumor immune response compared to that induced by cytoplasm-localized PDT (CP-PDT) [57]. (A) Chimeric peptide (PCPK, PpIX-C6-PEG8-KKKKKKSKTKC-OMe) targets cell PM through farnesyltransferase (PFTase) farnesylation. (B) PM-PDT-induced necrosis and ICD combined with aPD-1 therapy (Fig. 5a). The PCPK group with light trigger showed much more matured DCs than other groups (Fig. 5b). PCPK-SR and PCPK groups without light-triggered showed a lower amount of tumor infiltrating lymphocytes in tumors. After photo-irradiation, TIL proportion in the PCPK group was higher than that in the PCPG-SR group, which indicates that PM-PDT has a strong immune stimulation ability. In the light group, the TILs of PCPK + aPD-1 increased the most, indicating that PCPK-mediated PM-PDT can induce a stronger anti-tumor immune response, showing a more effective anti-tumor effect compared with the combination of aPD-1 therapy (Fig. 5c).

Oxygen-boosted PDT and PM-targeted PDT, like vascular-targeted PDT, achieve better immune response against tumors via enhancing the tumor-killing ability of PDT. As a result, PDT can be used alone or in combination with immunotherapy while its therapeutic effect on the tumor is enhanced. In this process, nanomaterials and nanotechnology play an important role in bridging the gap.

## Combination with immunotherapy

Although PDT may induce anti-cancer immunity, such effects usually are not sufficient to kill the remaining tumor and inhibit distant tumors [58]. To stimulate a strong anti-tumor immune response, a combination of therapy and multiple stimuli to expand immunogenicity is often necessary [59, 60]. Fortunately, the emergence of nanomaterials holds great potential to circumvent the disadvantages of the effect of PDT on metastatic tumors due to their unique properties.

### Nano-immuno-adjuvant photodynamic immunotherapy

To improve PDT-induced immune response, an effective strategy is to develop immune potentiator nanocapsules. Rose Bengal (RB), a staining substance and a sensitizer, is a suitable candidate for such application [61].

The combination of PDT and immune stimulation is also an effective strategy to enhance PDT-induced immune response. Nanoliposome encapsulating photosensitizer Chlorin e6 and Galectin-3 (Gal-3) inhibitor enhanced the ability of Natural Killer (NK) cells to recognize tumor cells after PDT [62]. 5-Aminolevulinic acid (5-ALA) is a novel photoactivator with high selectivity and can be turned into photosensitizer PpIX. Nanocomplexes can help 5-ALA bypass the skin barrier directly to the tumor to produce more ROS after PDT. T cells are highly enhanced to exploit host immunity to remove metastases from lymphoid organs, accompanied by a strong inflammatory response and destruction of the primary tumor [63].

**Fig. 6:**
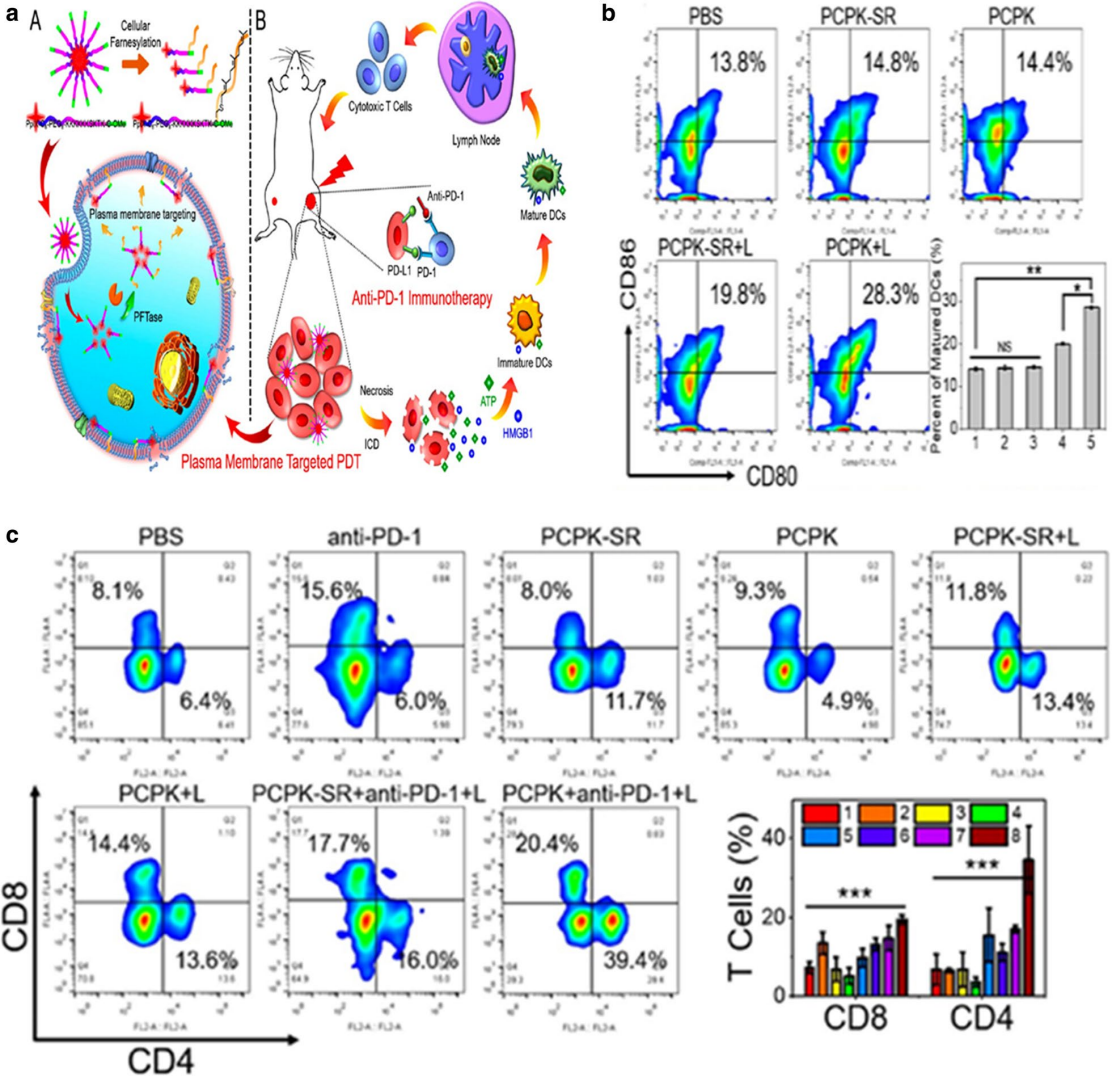
**a** Schematic diagram of neoantigen-based photodynamic immunotherapy. **b** Meanwhile, frequencies of T cells and DCs were analyzed **c** Tumor growth curves of each group[64] (Copyright © 2019 American Chemical Society)

Tumor Antigen is another immune enhancer that can be used in photodynamic immunotherapy. Xu et al. reported that mesoporous silica nanoparcicles (MSNs) with well-defined mesopores can successfully carry cancer vaccines to tumor sites avoiding rapid in vivo clearance [64]. PDT recruited dendritic cells and induced neoantigen-specific, tumor-infiltrating T lymphocytes. PDT combined with individual tumor inoculation has a strong synergistic effect and has a strong anti-tumor effect on both local and distant tumors. The authors used the heterogeneous oil–water double reaction system to synthesize bMSN. Ce6 and CpG were put into the mesopores of bMSNs, and after modification of bMSNs with PDP-PEG5k-NHS on the surface, the neoantigen peptide was conjugated to bMSNs. Laser irradiation (660 nm) was used to produce cytotoxic ROS, eliminate tumor cells, trigger local immune activation, and realize anti-tumor immunity (Fig. 6a). Compared with other groups, the bMSN vaccine + laser group induced more T cells and DCs (Fig. 6b). Compared with the untreated or laser-treated soluble vaccine group (P < 0.01), bMSN significantly delayed the growth of primary tumors. Importantly, PDT combined with the bMSN vaccine further slowed the growth of both primary and contralateral tumors (P < 0.05) (Fig. 6c).

### Combination with blockade therapy

Cancer immunotherapy has become an up-and-coming approach against cancer recently. Checkpoint blockade therapy has aroused great interest in recent years as promising immunotherapy for cancer [65]. However, Checkpoint blockade immunotherapy has been shown to benefit a minority of patients.

Checkpoint blockade immunotherapy only elicits limited a systemic anti-tumor response. PDT has shown the ability to induce a systemic anti-tumor immune response. Immunity-enhanced nano-PDT in combination with immune checkpoint inhibition might be a good strategy to eradicate both primary and metastatic tumors.

#### Combined with IDO inhibitor

Enzyme IDO is an immune checkpoint, which highly expressed in tumors to prevent the T cell cloning expression and promote T cell dysfunction and apoptosis.

**Fig. 7:**
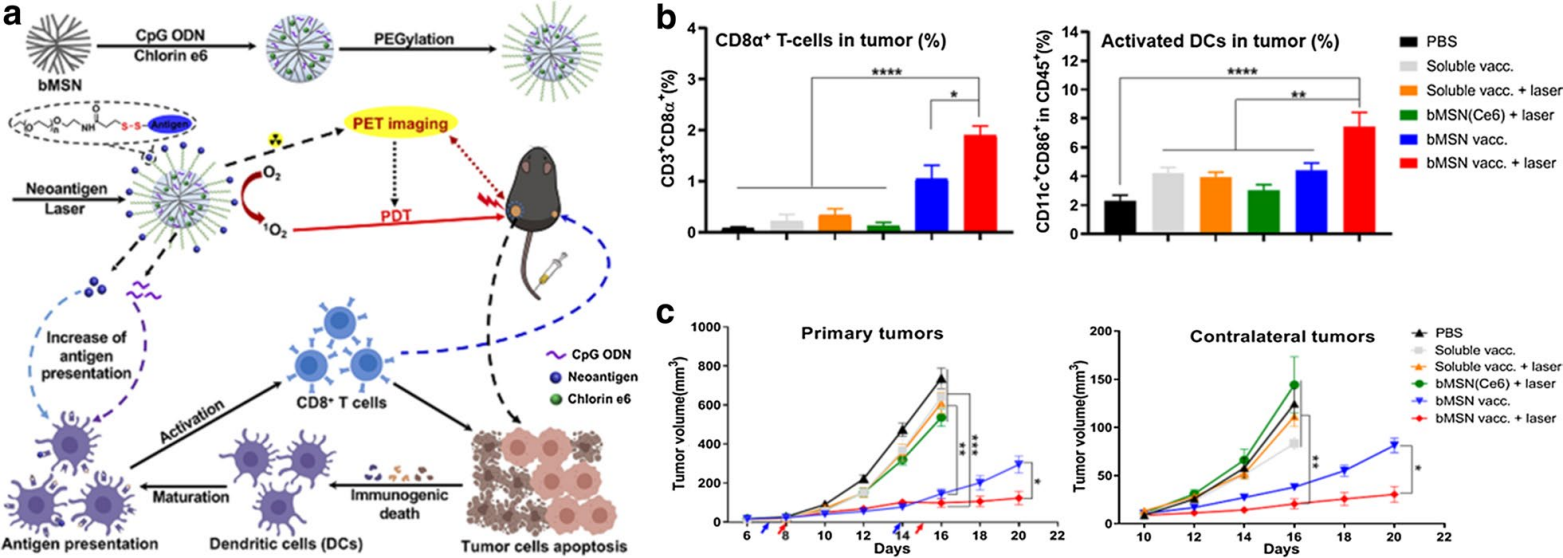
**a**Schematic diagram of PpIX-NLG@Lipo for PDT and IDO blockade combination therapy[66] Copyright © 2019 Ivyspring International Publisher. (b) Schematic diagram of combined PDT and immunotherapy using IND@RAL for combating cancer [67] Copyright© 2019 American Chemical Society. **c** A. The structure of PpIX-1MT. B. PDT leads to apoptosis and releasing of 1MT [68] (Copyright © 2018 American Chemical Society)

Tumor-associated antigens released by ICD induced by PDT can be presented to T cells when combining checkpoint blockade cancer immunotherapy [66–68]. First of all, PDT applying PPIX-NLG@LIPo could directly kill primary cancer cells. Meanwhile, PDT combined with indoleamine 2,3-dioxygenase (IDO) therapy could induce more CD8^+^T cells to proliferate and infiltrate in tumors(Fig. 7a) [66]. The RAL nanoplatform can accumulate in the tumor area through the EPR effect. After intratumoral IND@RAL laser irradiation, ROS are generated inside the tumor, causing apoptosis of tumor cells. Furthermore, the IDO inhibitor released by the IND@RAL nano-probes responded to the high-reduction environment, reversing the immunosuppressive TME (Fig. 7b) [67]. The chimeric peptide PpIX-1MT includes 3 parts. Palmitic acid plus PpIX are taken to fabricate the hydrophobic central part of self-assembled nanoparticles. The PEG segment was utilized to stabilize the molecular structure and form the hydrophilic shells. In this study, murine colon cancer cells were injected intravenously to establish a mice model of pulmonary timorous metastasis. It is supposed to accumulate in the primary tumor with a sturdy EPR effect through injection of PpIX-1MT nanoparticles, which could produce ROS under light irradiation to induce tumor cell apoptosis. Subsequently, the caspase-3 released by the tumor cells promotes the releasing of 1MT, causing CD8^+^T cell activation through inhibition of the IDO pathway. It has effective inhibition and eradication effect on both primary tumor and lung metastasis (Fig. 7c) [68].

**Fig. 8:**
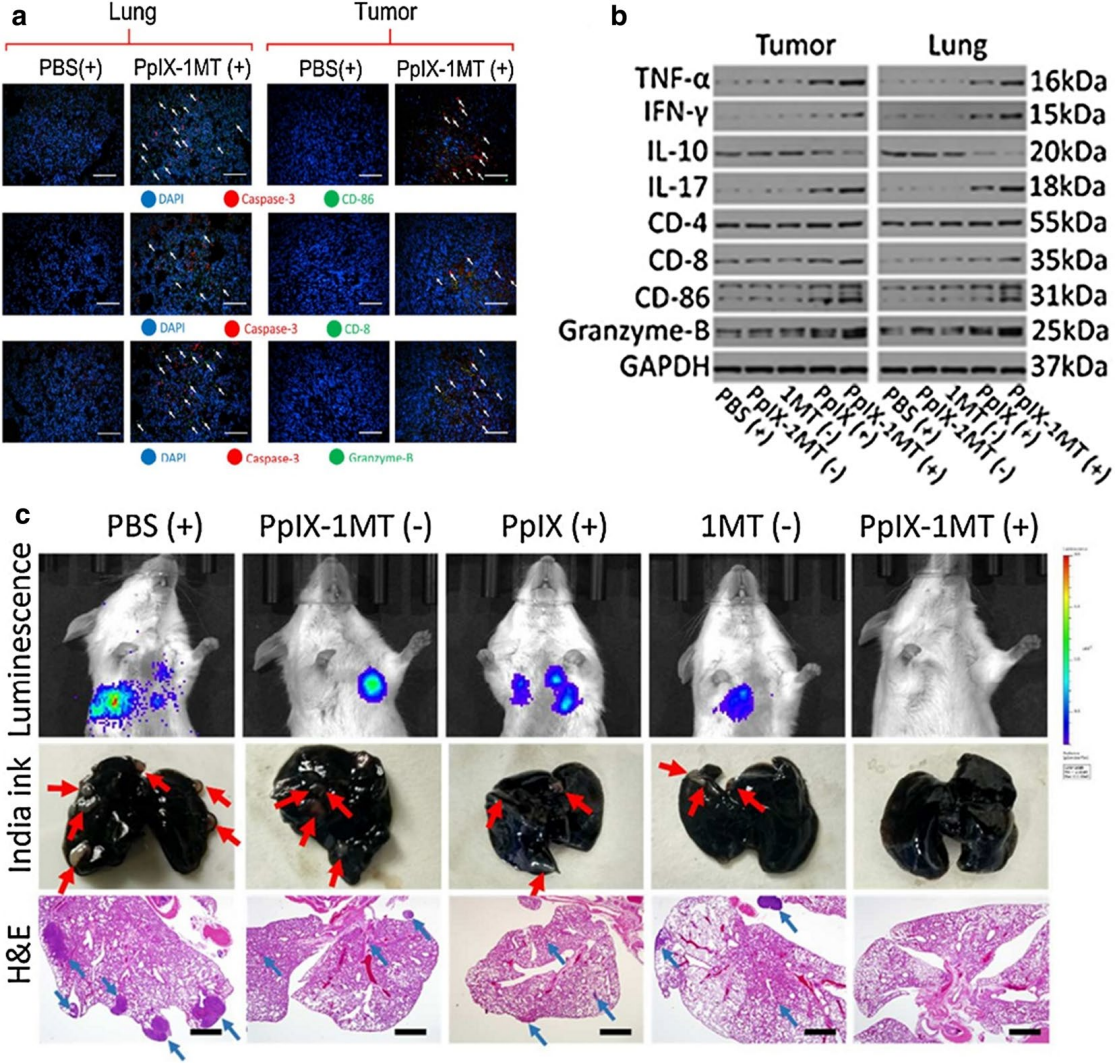
**a** Immunofluorescence of PBS and PpIX-1MT group. **b** Western blot analysis. **c** Evaluation of inhibition efficacy of lung metastasis [60] (Copyright © 2018 American Chemical Society)

An IDO inhibitor encapsulated in the nanoscale metal–organic framework (nMOF) increased T cell infiltration. Synergistic PDT and IDO checkpoint blockade therapy achieves primary and metastatic tumor inhibition [69]. Other two studies using the same IDO inhibitor loader liposome to achieve enhanced anti-tumor capability [61, 62]. Caspase responsive peptide can be utilized to combine the photosensitizer and IDO inhibitor. Cascade synergism can effectively inhibit the primary tumor and lung metastatic tumor [60]. Granzyme-B, CD8, CD86, and caspase-3 antibodies were used to label granzyme-B, CD8^+^T cells, DC cells, and dead cells respectively, to indicate the degree of immune response induced by each group. In comparison with PBS (+) group, the PpIX-1MT (+) group showed more granzyme-B, caspase-3, DC cells, and CD8^+^T cells in both primary tumor and lung (Fig. 8a). The immune-facilitate indexes include TNF-α, interferon-gamma (IFN-γ), interleukin-17(IL-17), CD-8, CD-86, granzyme-B were ascending from PBS (+) group to PpIX-1MT (+) group (Fig. 10b). While the IL-10, a marker of immune suppression, showed the opposite tendency (Fig. 8b). Compared with other groups, no luminescence was found in the PpIX-1MT (+) group. By comparison, lung tissues from the other groups showed strong luminescence. The PpIX-1MT (+) group had almost no pulmonary tumorous nodules, while the other groups had a lot of pulmonary tumorous nodules (red arrow indicates lung tumor nodules). In particular, white tumorous nodules appeared in PpIX (+) group and 1MT (−) group, suggesting that neither PDT alone nor 1MT alone could eradicate pulmonary metastasis. H&E staining of lung tissues was further to be confirmed (Fig. 8c).

#### Combined with CTLA-4 checkpoint blockade therapy

**Fig. 9:**
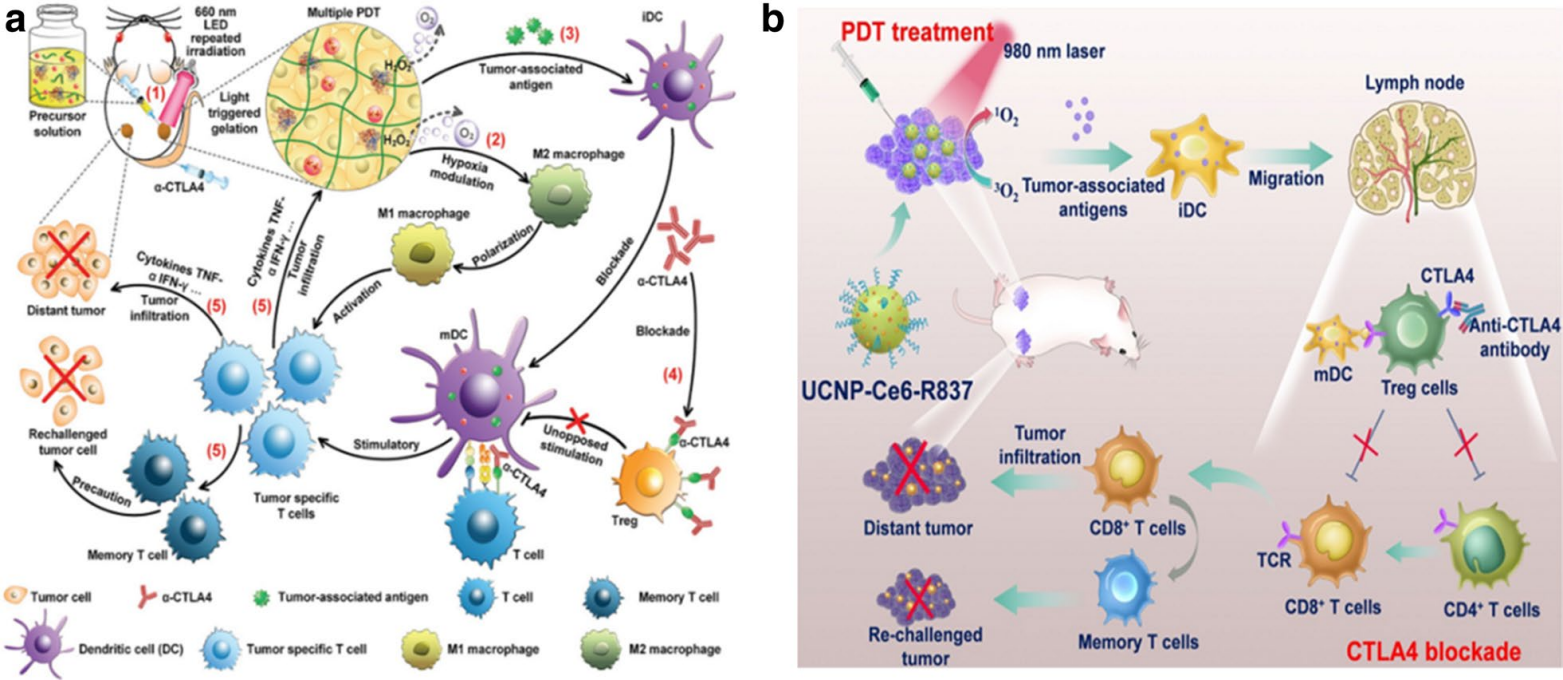
**a** This protocol elucidates the mechanism by which hydrogel-based photodynamic immunotherapy achieves systemic immune response. (1) The in situ gel is triggered by light so that the injected components can be maintained for a long time. (2) Remission of tumor hypoxia by catalase can enhance PDT and immunotherapy. (3) Tumor fragments and nano-adjuvant nanoplatform can induce a strong immune response. (4) Anti-tumor immunity can be further enhanced by checkpoint blockade that controls immune balance. (5) Under these synergistic mechanisms, gelatin-based PDT immunotherapy can effectively eliminate local, distal, and recurrent tumors[70] Copyright © 2019 John Wiley & Sons, Inc. **b** The mechanism of combined application of NIR-PDT and anti-CTLA 4 therapy was summarized. UCNP-Ce6-r837 nanoparticles can effectively perform photodynamic destruction on tumors under near-infrared light. Generated by the presence of tumor-associated antigen these nanoparticles as adjuvants can inhibit both primary and distant tumors and produce remaining immune memory [58] (Copyright © 2017 American Chemical Society)

Multi-round PDT based on hydrogel in combination with anti-CTLA 4 therapy can not only inhibit the growth of the distant tumor, but also effectively protect immune memory and prevent tumor recurrence in a relatively long time [70]. The mechanism of hydrogel-based photodynamic immunotherapy (Fig. 9a). Immunoadjuvant nanoparticles can promote a strong anti-tumor immunity in the presence of tumor-associated antigens produced by tumor photodynamic destruction. Thereby, immune adjuvant PDT combined with anti-CTLA 4 therapy has a good effect on the tumor under near-infrared laser irradiation and inhibits metastatic tumor growth after PDT [58]. The underlying mechanism of immune memory against tumor recurrence are as follows (Fig. 9b).

#### Combined with PD-1 and PD-L1 blockade therapy

**Fig. 10:**
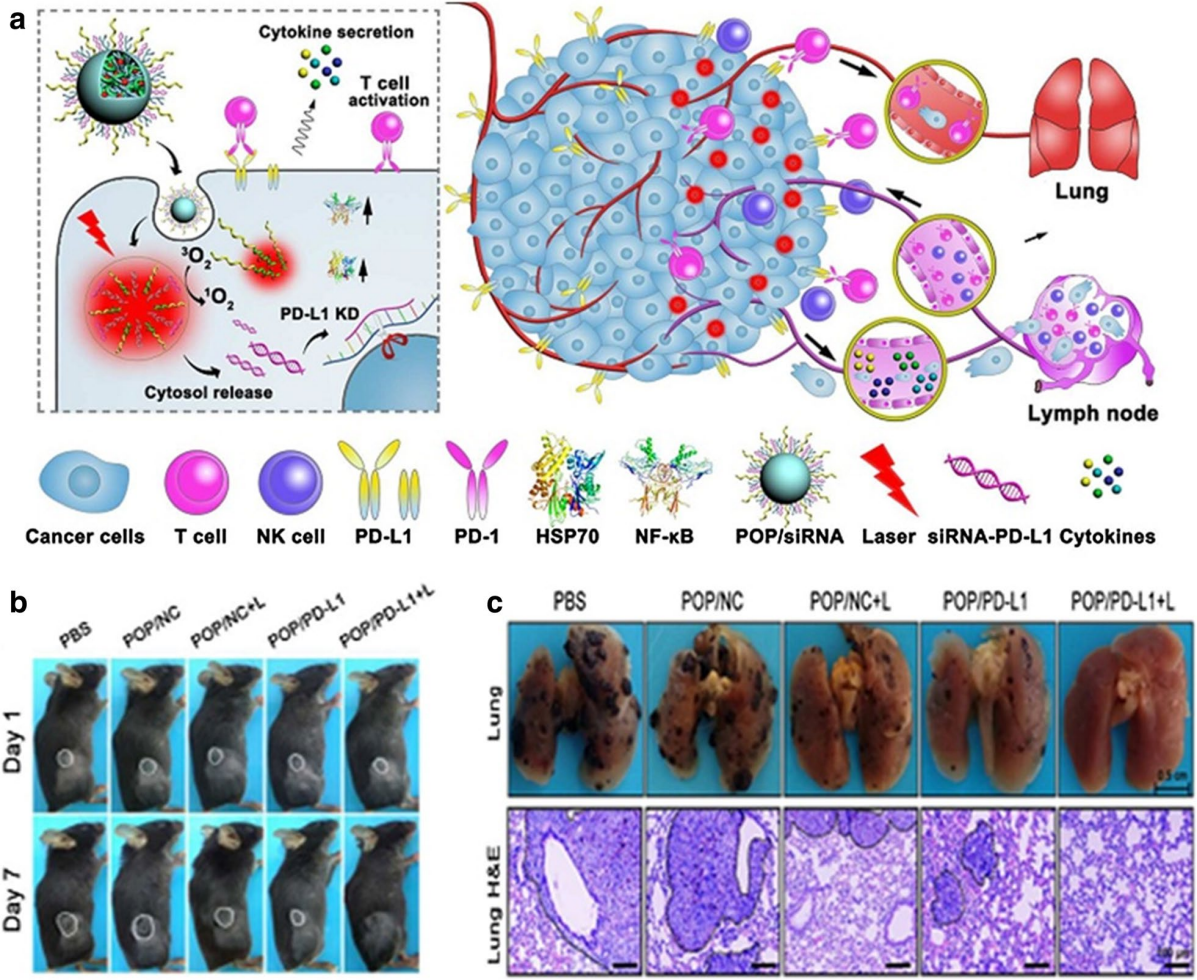
**a** Schematic illustration acid-activated multifunctional micelleplexes are used in cancer photodynamic immunotherapy with PD-L1 blockade enhancement. **b** Photos of mice taken at first or seventh day after PDT and PD-L1-KD. **c** Images of metastatic foci [71] (Copyright© 2016 American Chemical Society)

Wang et al. reported that the acid-activatable small interfering RNA (siRNA) micelleplexes combining PDT and aPD-L1 significantly enhanced the inhibition of primary and metastatic tumors (Fig. 10a). PDT or PD-L1 knockdown (KD) alone can attribute to tumor growth inhibition of ~ 73% and ~ 65%, respectively. It is worth noting that PDT and PD-L1 KD combined therapy fully abolished tumors with no weight loss (Fig. 10b). PDT combined with PD-L1 KD significantly inhibited metastatic lung tumor (Fig. 10c) [71].

**Fig. 11:**
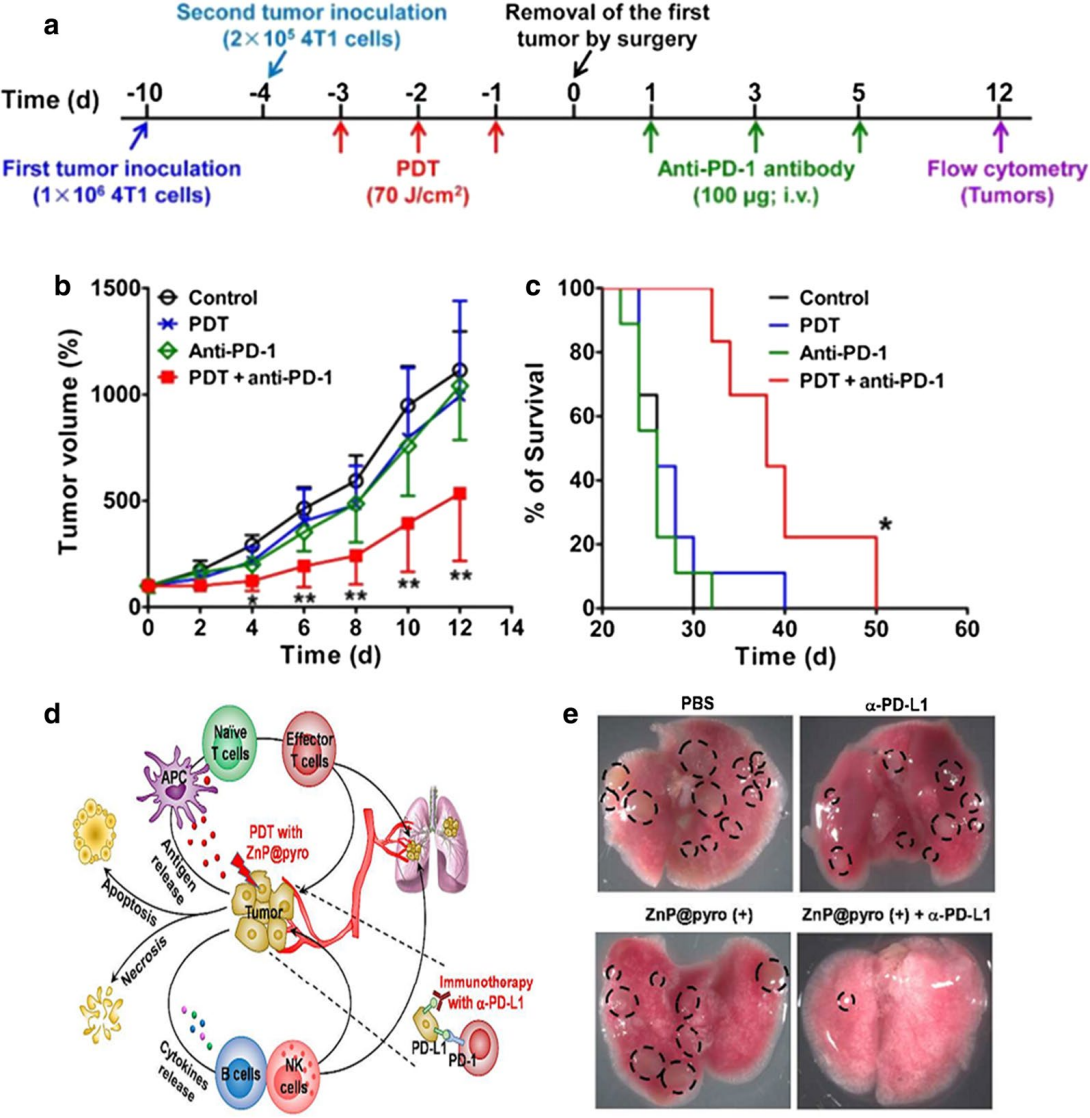
**a** diagram of DSAB-HK PDT combined with aPD-1 therapy. **b**, **c** The growth curves of the second tumors (**b**) and survival curves of the mouse (**c**) after treatments and removal of first tumor [72] Copyright © Ivyspring International Publisher. **d** ZnP@pyro PDT combined with aPD-L1 therapy can eradicate both primary and distant tumors via anti-tumor immunity. **e** Typical images of the nodules in the lung neoplasm are shown [73] (Copyright© 2016 American Chemical Society)

Targeting PDT promotes dendritic cell maturation and cytokine production, thereby stimulating activation of CD8^+^ cytotoxic T lymphocytes. Therefore, Yu et al. reported that combining with aPD-1 therapy significantly inhibited second subcutaneous tumor and metastasis [72]. The scheme of DSAB-HK PDT combined with aPD-1 therapy (Fig. 11a). aPD-1 therapy after DSAB-HK PDT significantly inhibited the second tumor growth (Fig. 11b). aPD-1 therapy after DSAB-HK PDT significantly prolonged survival (Fig. 11c). Combined with aPD-1 therapy, the inhibitory activation of the immune response caused by PDT also inhibited distant tumors [60].

Nanoparticle-mediated PDT that generating T cells enhances the systemic efficacy of aPD-1 therapy in primary tumors and lung metastases [73]. Combination therapy of checkpoint blockade therapy and routine immunogenic therapy like PDT can significantly increase the ratio of tumor response to checkpoint therapy (Fig. 11d). Compared with the PBS control group, ZNp@Pyro PDT or anti-PD-L1 therapy alone showed little effect in preventing lung metastasis, while combination therapy significantly reduced tumor nodules (Fig. 11e).

**Fig. 12:**
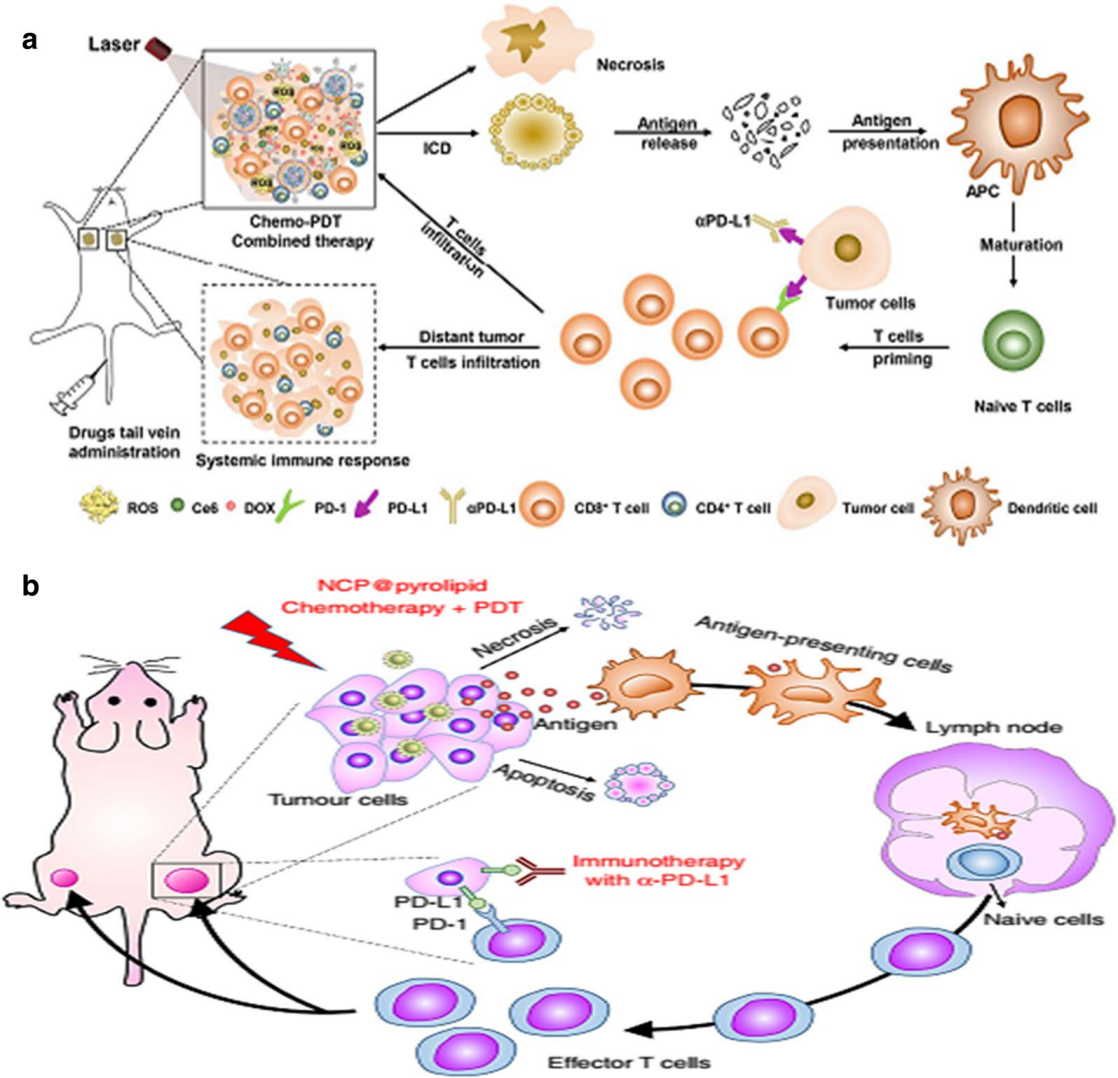
**a** The mechanism of TKHNPC/D-mediated chemo-PDT combined with aPD-L1 therapy to induce anti-tumor immune response[75] Copyright © 2019 Elsevier Ltd. **b** Chemo-PDT of NCP@pyrolipid can enhance the aPD-L1 therapy and cause systemic anti-tumor immunity [74] (Copyright © 2016 Springer Nature Limited)

Chemotherapeutic drugs can enhance PDT evoked anticancer immune responses. Combined with aPD-L1 therapy, chemotherapeutic drugs loaded nanoparticles can both inhibit primary and distant tumor growth [74, 75]. Under 660 nm irradiation, encapsulated Ce6 produced ROS, which could induce PDT for cancer treatment and rapidly degraded TK-PPE, promoted the release of intracellular DOX, induced efficient chemotherapy-PDT cascade, and inhibited tumor growth through monotherapy. The cascaded chemo-PDT could lead to DOX-based chemotherapy-PDT induced ICD. Cascaded chemo-PDT further combined with aPD-L1 therapy effectively inhibits the growth of primary and cause distant tumors to degenerate through significant distal effects, demonstrating the enhanced effectiveness of checkpoint blockade antibodies (Fig. 12a) [75]. Combined with aPD-L1 therapy, NCP@pyrolipid chemo-PDT greatly promotes the generation and infiltration of tumor-specific effecter T cells in tumors, resulting in the elimination of the main tumor site and a systemic anti-tumor immune response rejecting remote tumor (Fig. 12b) [74].

## Conclusions and future challenges

Owing to the extremely short diffusion range of singlet oxygen, it would be more effective to deliver photosensitizers directly to specific organelle to overcome hypoxia-induced PDT resistance. Nanomaterials that can regenerate and reperfuse O_2_ can be used to deal with tumor hypoxia too. PDT can cause local damage to the tumor by direct cytotoxic effects and vascular damage, as well as inflammatory response. But the anti-tumor effects of PDT are usually inefficient in the complete eradication and long-term control over disseminating tumors. Supported by nanomaterials augmented systemic anti-tumor immunity, nanotechnology-based PDT destroys both primary and metastatic tumor in many types of tumors.

PDT is seldom employed in deep-seated tumors on account of its poor tissue penetration by light. High penetrating microwave or X-ray irradiation of PSs can solve this problem. MW can induce heat to increase blood supply to enhance the PDT effect. The X-ray can also overcome the limits of tissue attenuation. X-ray activates PSs to generate ROS, which is used for X-ray-induced PDT. However, X-ray may be attenuated by hypoxia, this means more modalities are needed to yield X-ray and PDT synergistic anti-tumor effects. PSs cannot be activated directly by X-ray, so nanomaterials can be used to bridge the gaps and overcome the hypoxia in the tumor. Since X-ray is harmful to normal tissue, making X-ray-induced PDT less selective. Efforts should be focused on fabricating the best nanomaterials with fewer X-rays. Gas therapy is a novel treatment utilizing gas, including CO, NO, and H_2_S. PDT is oxygen-consuming and tumor hypoxia reduces the therapy efficiency. Small molecule NO has a far diffusion radius and long half-life in the hypoxic tumor microenvironment. Part of the ROS generated by PDT can play an oxidative role in transforming NO donors into NO, which significantly enhances the effect of PDT. Gas therapy and PDT can become an organic combination to achieve collaborative treatment [76].

There have been numerous advancements in immunotherapy. However, immunotherapy alone is not effective for the treatment of cancer. In the combinational treatment of PDT and immunotherapy, PDT can target primary tumors that can not be treated by immunotherapy. Nanotechnology can be helpful to develop multimodal immunotherapy strategies for cancer therapy. The relationship between the human immune system and disease is complex and individually unique, which makes the immunotherapy effect compromised and difficult to predict. Immunotherapy combined with PDT base on nanotechnology might provide a more specific and hopeful treatment to fight the tumor. However, PDT-induced immunotherapy is badly impaired by T cell activities through the PD-1/PD-L1 immune checkpoint pathway. Compared with surgery or chemotherapy, both photodynamic and immunotherapy are minimally invasive treatments with lower side effects. But the effect of photodynamic therapy on the tumor is not as good as that of surgery and chemotherapy, which makes the effect of photodynamic immunotherapy on solid tumors questionable.

The tumor-targeting ability of PDT is significantly limited by the tumor hypoxic microenvironment. Nanomaterials-based photodynamic immunotherapy by conjugating nanomaterials with tumor-targeted molecules and antibodies could improve the targeting ability of PDT. Nano-PDT can induce an immunogenic TME, which makes tumors sensitive to checkpoint blockade therapy. Nano-PDIT can inhibit metastatic tumors and have a long-term immune memory. Checkpoint blockade immunotherapy is very efficient but beneficial to only a small number of patients because of an inadequate immune response. When combining with nano-PDT, checkpoint blockade therapy can kill primary and metastatic tumors at the same time by activating host immune systems.

Malignant brain cancers usually recur despite standard treatment of surgery followed by radiation therapy or chemotherapy, making it one of the most difficult challenges in cancer treatment. And PDT is a treatment option for brain cancer. Patients with recurrent anaplastic astrocytoma or with recurrent glioblastoma multiforme both had an extended median survival time after PDT [77]. Except for intraoperative PDT, X-ray-induced PDT is a more minimally invasive treatment for brain cancer. Although the brain is not a part of systemic immune reactions, there is still some immunosuppression in brain tumors for immune protection [78]. Therefore, nano-Photodynamic immunotherapy may be applied to brain cancer treatment for nanomaterials that can be utilized to control antigen and adjuvant delivery. But brain toxicity of nanomaterials needs to be studied fully.

PDT and photothermal therapy (PTT) induces oxidative stress and thermal stress in tumor cell respectively. ROS induced by PDT can strengthen the immune response. Different from PDT, PTT causes tumor cell death by heat to release tumor-associated antigens into the surrounding environment to produce an anti-tumor response [79]. Nanomaterials can then be applied to enhance the anti-tumor immunity of PDT and PTT accordingly. Since PDT procedures require sufficient oxygen, PTT can increase oxygen in the tumor by improving blood flow, combined PTT-PDT immunotherapy might be a promising anti-tumor treatment option. Although nanomaterials can achieve PTT-PDT, they still suffer from unclear toxicity.

Although numerous preclinical studies have shown that photodynamic immunotherapy based on nanotechnology enhances immunity against primary and metastatic tumors simultaneously. However, this effect of nanotechnology-based photodynamic immunotherapy has not been examined clinically. Photodynamic therapy has clinically shown a good effect in the treatment of tumors of the body surface and cavity organs. Due to the limited tissue penetrating ability of light, for large solid tumors such as hepatocellular carcinoma, photodynamic immunotherapy can be performed by using hollow needles to puncture and insert photodynamic fibers multiple times.

The safety of nanomaterials is a major future challenge for nano-photodynamic immunotherapy. However, for the safety of nanomaterials, cell and animal experiments are mainly carried out at the present stage, with few human experimental data. However, there are many kinds of nanomaterials, so it is necessary to carry out a lot of human toxicity experiments before they can be used in clinical practice.

## Data Availability

Not applicable.
